# Nuclear envelope tethering inhibits the formation of ALT-associated PML bodies in ALT cells

**DOI:** 10.18632/aging.202810

**Published:** 2021-04-04

**Authors:** Chia-Wei Yang, Meng-Hsun Hsieh, Hao-Jhe Sun, Shu-Chun Teng

**Affiliations:** 1 Department of Microbiology, College of Medicine, National Taiwan University, Taipei 10051, Taiwan; 2 Center of Precision Medicine, National Taiwan University, Taipei 10051, Taiwan

**Keywords:** alternative lengthening of telomeres, telomere-telomere recombination, nuclear envelope tethering, SUN1, RAP1

## Abstract

Telomere length homeostasis is essential for maintaining genomic stability and cancer proliferation. Telomerase-negative cancer cells undergo recombination-mediated alternative lengthening of telomeres. Telomeres associate with the nuclear envelope through the shelterin RAP1 and nuclear envelope SUN1 proteins. However, how the associations between telomeres and the nuclear envelope affect the progression of telomere recombination is not understood. Here, we show that telomere anchorage might inhibit telomere-telomere recombination. SUN1 depletion stimulates the formation of alternative lengthening of telomeres-associated promyelocytic leukemia bodies in ALT cells. In contrast, overexpression of a telomere-nuclear envelope-tethering chimera protein, RAP1-SUN1, suppresses APB formation. Moreover, inhibition of this nuclear envelope attachment alleviates the requirement of TOP3α for resolving the supercoiling pressure during telomere recombination. A coimmunoprecipitation assay revealed that the SUN1 N-terminal nucleoplasmic domain interacts with the RAP1 middle coil domain, and phosphorylation-mimetic mutations in RAP1 inhibit this interaction. However, abolishing the RAP1-SUN1 interaction does not hinder APB formation, which hints at the existence of another SUN1-dependent telomere anchorage pathway. In summary, our results reveal an inhibitory role of telomere-nuclear envelope association in telomere-telomere recombination and imply the presence of redundant pathways for the telomere-nuclear envelope association in ALT cells.

## INTRODUCTION

Telomeres protect the ends of linear chromosomes from end-to-end fusion, injurious chromosomal rearrangements, and genomic instability [1]. Human telomeric DNA is composed of a serial of hexameric DNA repeats (5’-TTAGGG-3’) that are synthesized by telomerase, an RNA-protein complex [2, 3]. In normal conditions, the telomeres of most human somatic cells are continuously shortened during each cell division due to the absence of telomerase and incomplete replication. When telomeres become extremely short, they lose protection abilities and are recognized as broken-ends, which turn on cell cycle checkpoint signaling. In human fibroblasts, gradually losing telomeres induce chromosome fusion, crisis, and apoptosis [4]. Cancer cells can circumvent this crisis through the reactivation of telomerase (85%) or the alternative lengthening of telomeres [5] recombination pathway (15%) to elongate their telomeres [6–8]. Many DNA repair and recombination proteins have been discovered in the ALT pathway, but the molecular mechanisms and regulations of ALT are still not fully understood [6, 9].

ALT cells possess a unique marker, ALT-associated promyelocytic leukemia (PML) bodies (APBs), containing telomeric DNA as well as many DNA repair and replication proteins, such as RAD52, RAD51, RAD50, RPA, TRF1, TRF2, and NBS1 [10]. Many studies speculated that APBs provide a ‘‘recombinogenic microenvironment’’ to benefit ALT formation [11–19]. APBs can be detected within a mitotic cell cycle [20], and seem to be dynamic in the nucleus [19–22]. High-resolution images revealed that APB-associated telomere clustering promotes telomere-telomere recombination after replication [23]. A recent study showed that telomeric DNA synthesis exclusively occurs in APBs [18]. In addition to APBs, another characteristic of ALT cells is the presence of the high content of extrachromosomal telomeric DNA circles, especially single-stranded C-rich circles (C-circles) [14, 24–30]. Nonetheless, how the C-circles are generated remains mysterious.

The telomere-associated shelterin proteins TRF1, TRF2, TIN2, and RAP1 act as negative regulators of telomere length in telomerase-positive cells to assure the telomeric integrity [31]. They are also important for APB formation [10, 32, 33]. Knockout mice studies showed that loss of RAP1 increases the frequency of telomeric sister chromatid exchanges (T-SCEs) in mouse embryonic fibroblasts (MEFs), suggesting that RAP1 is critical for the repression of telomeric recombination in MEFs [34, 35]. However, the mechanism of how shelterin RAP1 regulates telomere-telomere recombination in ALT cancer cells remains to be clarified.

Linkers of nucleoskeleton and cytoskeleton (LINC) complexes are conserved nuclear membrane proteins that span in the nuclear envelope to connect cytoskeletal and nucleoskeletal elements [36, 37]. The LINC complexes play critical roles in multiple fundamental cellular processes, including nuclear migration, meiotic chromosome pairing, mechanotransduction, and nuclear shape maintenance [38]. The LINC complexes consist of the conserved inner nuclear membrane SUN proteins and outer nuclear membrane KASH proteins [36]. They are involved in the chromatin mobility, mobility, and clusters of DNA breaks, and double-strand break repair [39–43]. SUN1 and SUN2 were the first identified SUN proteins that are widely expressed in mammals [44–47]. The N-terminal domain of SUN1 locates in the nucleoplasm, while its C-terminal domain is inserted into the perinuclear space for docking the KASH proteins [36, 37]. Immunohistological data showed that SUN1 expression was reduced in breast cancer tissues and cell lines as compared to the normal mammary gland tissues, suggesting that reduction of SUN1 plays a pathological role in breast cancer formation [48]. SUN1 is also important for messenger RNA export, nuclear pore complex distribution, and nucleolar morphogenesis [49–52].

Telomeres of T-lymphocytes are positioned within the interior 50% of the nuclear volume [53]. Other studies showed that telomeres seem to attach to the nuclear matrix during interphase in non-ALT cells [54–56]. Surprisingly, it was later revealed that telomeres can attach to the nuclear envelope during the mitotic cell cycle through the interaction between RAP1 and SUN1 in HeLa and IMR90 cells [57]. On the contrary, telomere tethering to the nuclear envelope in ALT cells has not been examined. We were curious about whether telomere tethering affects the telomere-telomere recombination pathway in ALT cells.

In this study, we found that depletion of the nuclear envelope SUN1 promotes the APB formation and C-circle levels in ALT cells. On the other hand, the APB formation is impaired when the telomeres are forced to link with the nuclear envelope, suggesting that the telomere-nuclear envelope attachment may prevent telomere-telomere recombination. SUN1 knockdown recovers the deficiency of the APB formation in the TOP3α-depleted cells, suggesting that the reduction of the nuclear membrane tethering may partially relieve the requirement of TOP3α during telomere-telomere recombination. Moreover, our data imply a SUN1-dependent but RAP1-independent telomere-tethering pathway that may also prevent telomere-telomere recombination in ALT cells.

## MATERIALS AND METHODS

### Cell culture, transfections, and methionine treatments

HEK-293T, U2OS, VA13, HeLa, and HCT116 cells were cultured in Dulbecco’s modified Eagle’s medium (DMEM) supplemented with 10% fetal bovine serum, penicillin, streptomycin, and nonessential amino acids (HyClone). Transfection was conducted using T-Pro nonliposome transfection reagent II (T-Pro Biotechnology). To increase the APB formation ratio, virus-infected U2OS and VA13 cells were treated with methionine restriction as previously described [33]. In brief, cells were grown on glass coverslips in DMEM to 50% confluency. The cells were washed once with methionine-deficient medium before incubation in methionine-deficient medium for 3 days; this methionine-deficient DMEM (Gibco) contained 10% fetal bovine serum, penicillin, streptomycin, 4 mM glutamine, nonessential amino acids (HyClone), and L-cysteine (48 mg/l, Sigma).

### Viral infection

HEK-293T cells were cotransfected with the packaging plasmid (pCMV-Δ8.91), envelope (pMD.G), and either hairpin pLKO-RNAi vectors or cDNA expression pLAS5w.Pneo vectors (National RNAi Core Facility, Institute of Molecular Biology/Genomic Research Centre, Academia Sinica, Taiwan) for virus production. The specific oligo sequences of the shRNA are listed in Supplementary Table 1. Twenty-four hours posttransfection, the medium was replaced with DMEM containing 1% BSA. Virus-containing supernatants were collected at 48 h and 72 h posttransfection. The cells were infected with viruses and cultured in DMEM containing 1 μg/ml polybrene (Millipore) for 16 h. The transduced cells were selected with DMEM containing 1 μg/ml puromycin (Invitrogen) or G418 (Sigma) for the indicated days (600 μg/ml G418 for VA13 cells and 800 μg/ml G418 for U2OS cells).

### Plasmid construction

The primers used in this study are listed in Supplementary Table 2. For pcDNA3HA-RAP1 construction, *RAP1* cDNA was amplified from U2OS cell cDNA by PCR using the primers RAP1-BamHI-1F and RAP1-XhoI-R and then cloned into the BamHI-XhoI sites of pcDNA3HA (a gift from Dr. Tsai-Kun Li). For pLAS5w-RAP1 construction, *RAP1* cDNA was amplified by PCR using the primers RAP1-NheI-For and RAP1-NsiI-Rev and then cloned into the NheI-NsiI sites of pLAS5w.Pneo (National RNAi Core Facility, Institute of Molecular Biology/Genomic Research Centre, Academia Sinica, Taiwan). The pcDNA3HA-RAP1 and pLAS5w-RAP1 truncated mutants were generated using a QuickChange site-directed mutagenesis kit (Stratagene) with the primers listed in Supplementary Table 2. The pEGFP-SUN1 N205 plasmid was generated by self-ligation of SmaI-digested pEGFP-SUN1 (a gift from Dr. Angelika A. Noegel), which removes the SUN1 C-terminus. For pLAS5w-SUN1 construction, *SUN1* cDNA was amplified from pEGFP-SUN1 by PCR using the primers NheI-SUN1-For and SUN1-EcoRI-Rev and then cloned into the NheI-EcoRI sites of a pLAS5w.Pneo plasmid. The pLAS5w-RAP1-SUN1 fusion was constructed as follows: the *RAP1*-G8 sequence was amplified by PCR using the primers HpaI-RAP1-For and RAP1-G8-NheI-Rev, which contain the sequence for eight glycine residues before the NheI site at the 3’ end of the reverse primer, and then cloned into HpaI-NheI in pLAS5w.Pneo to create pLAS5w-RAP1-G8. Next, *SUN1* cDNA was amplified by PCR using the primers NheI-SUN1-For and SUN1-EcoRI-Rev and inserted into the NheI-EcoRI sites of pLAS5w-RAP1-G8 to obtain pLAS5w-RAP1-SUN1. To create RAP1 ΔC, which cannot interact with TRF2 [58], RAP1 amino acid 1-289 sequences were amplified by PCR using the primers HpaI-RAP1-For and RCTdel-G8-NheI-Rev. The 3’ end of the reverse RCTdel-G8-NheI-Rev primer contains the sequence for eight glycine residues before the NheI site. The PCR-amplified HpaI- and NheI-digested RAP1 ΔC fragments were cloned into pLAS5w-SUN1 to obtain pLAS5w-RAP1ΔC-SUN1. The RAP1 8FA and RAP1 8DE sequences were synthesized by gBlocks^®^ gene fragments (Integrated DNA Technologies) and then cloned into the pcDNA3HA and pLAS5w plasmids, respectively. All plasmids were sequenced before use.

### Cell fixation and immunofluorescence assays

Cells were seeded on glass coverslips coated with 0.2% gelatin (Sigma). After washing with phosphate-buffered saline (PBS), the cells were fixed in 4% paraformaldehyde in PBS for 10 min at room temperature. The fixed cells were permeated with 0.05% Triton X-100 in PBS for 5 min. These cells were washed three times with PBS and incubated with 1% BSA in PBS for 1 h at room temperature. For immunostaining, cells were incubated for 2 h with anti-TRF2 (1:200, clone 4A794, Millipore) and anti-PML (1:150, N-19, sc9862, Santa Cruz Biotechnology) primary antibodies at room temperature. The cells were washed twice with PBS for 5 min each time and then incubated with chicken anti-goat Alexa Fluor 594 (Invitrogen) for 1 h at room temperature. The cells were washed twice with PBS and incubated with goat anti-mouse Alexa Fluor 488 (Invitrogen) and 4',6-diamidino-2-phenylindole (DAPI) (Sigma) for 1 h at room temperature. The cells were washed three times with PBS and mounted on glass slides. Immunofluorescence images were acquired with a Zeiss Axioplan fluorescence microscope.

### Immunoblotting

Cell lysates were prepared in sample buffer, separated by sodium dodecyl sulfate-polyacrylamide gel electrophoresis (SDS-PAGE), and transferred to a PVDF membrane. The primary antibodies used in this study were anti-GAPDH (GTX100118, GeneTex), anti-Lamin A/C (N-18, sc-6215), anti-SUN1 (EPR6554, ab124770, Abcam), anti-RAP1 (A300-306A, Bethyl Laboratories), and anti-TOP3α (14525-1-AP, Proteintech). Horseradish peroxidase (HRP)-conjugated sheep anti-mouse and donkey anti-rabbit antibodies (GE Healthcare) were used as secondary antibodies. Immunoreactivity was detected by chemiluminescence using X-ray film (Fujifilm Corporation).

### C-circle assay

The C-circle assay was performed as previously described [26]. Briefly, each DNA sample (10 and 40 ng) was incubated with or without 7.5 U ɸ29 DNA polymerase (NEB, M0269) in a 20-μl reaction mixture containing 9.25 μl of 2.16x master mix (8.65 mM DTT, 2.16x ɸ29 buffer, 8.65 μg/ml BSA, 0.216% v/v Tween 20, 2.16 mM dATP, 2.16 mM dGTP, 2.16 mM dTTP, and 2.16 mM dCTP) for performing the rolling circle amplification reaction at 30° C for 8 h and then transferred to 70° C for 20 min to inactivate ɸ29 DNA polymerases. The reaction mixture without the addition of ɸ29 DNA polymerase was used as a control. The reaction mixtures were loaded onto an Amersham Hybond-N+ membrane using the Bio-Rad Bio-Dot system. The membrane was cross-linked with 454 nm UV-C at 1,200 J twice and hybridized overnight with a ^32^P-labeled (Invitrogen) CCA oligonucleotide probe (5’-CTAACCCTAACCCTAACC-3’) in hybridization buffer (225 mM NaCl; 15 mM NaH_2_PO_4_; 1.5 mM EDTA, pH 7.6; 10% polyethylene glycol 8000; and 7% SDS) at 37° C. The membrane was washed three times with washing solution (0.5 × SSC, 0.1% SDS) and exposed to X-ray film (Fujifilm Corporation). The images were quantified using NIH ImageJ software.

### Immunoprecipitation

Cells were lysed in lysis buffer (150 mM NaCl; 1% Triton X-100; 50 mM Tris-HCl, pH 8.0; 1 mM PMSF; Roche protease inhibitor; and a phosphatase inhibitor cocktail) on ice for 10 min. After centrifugation at 13,000 rpm for 10 min at 4° C, 1 mg of protein lysate was incubated with 50 μl of μMACS anti-GFP microbeads (Miltenyi Biotec) for 1 hour at 4° C. Labeled proteins were applied to a μMACS column placed in a μMACS separator (Miltenyi Biotec). Then, the column was washed three times with Wash Buffer 1 (150 mM NaCl, 1% NP-40, 0.5% sodium deoxycholate, 0.1% SDS, and 50 mM Tris-HCl, pH 8.0) and further washed once with 20 mM Tris-HCl, pH 7.5. The target proteins were eluted in 50 μl elution buffer preheated to 95° C (50 mM Tris-HCl, pH 6.8; 50 mM DTT; 1% SDS, 1 mM EDTA; 0.005% bromophenol blue; and 10% glycerol) for SDS-PAGE analysis.

### Telomere restriction fragment (TRF) analysis

Genomic DNA was extracted from cells by a genomic DNA purification kit (Promega). Genomic DNA (2 μg) was digested with RsaI and HinplI (New England Biolabs), and run on a 0.5% UltraPure^TM^ Agarose gel (Invitrogen) in 0.5 × TBE buffer using a Thermo Scientific ^TM^ Owl ^TM^ A1 Large Gel System (Invitrogen) at 120 V for 20 h. The gel was soaked in 0.25 N HCl for 15 min to denature the DNA and then neutralized in a 0.5 N NaOH and 1.5 M NaCl solution for 30 min. The digested DNA was transferred onto a Hybond N+ nylon membrane (GE healthcare). The membrane was cross-linked twice with 454 nm UV-C at 1,200 J twice and hybridized overnight with a ^32^P-labeled (Invitrogen) 800-bp TTACCC telomere-specific probe in Church buffer (1% BSA, 1 mM EDTA, 0.5 M phosphate buffer, and 7% SDS) at 65° C. The next day, the blot was washed three times with 4 × SSC buffer and then exposed to X-ray film (Fujifilm Corporation). Telomere length was determined by ImageQuant TL software (GE healthcare).

### Statistical analysis

Each experiment was repeated at least three times. The results are expressed as the means ± standard deviation (SD). A two-tailed Student’s t-test was used for statistical analysis. *P* < 0.05 was considered statistically significant.

## RESULTS

### Depletion of SUN1 promotes APB and C-circle formation in ALT cells

The role of SUN1 in telomere-nuclear envelope anchorage in non-ALT cells [57] prompted us to investigate whether SUN1 is required for telomere-telomere recombination in ALT cells. We first examined the significance of SUN1 in the viability of U2OS and VA13 ALT cells. Depletion of SUN1 slowed the growth of both ALT cell lines (Supplementary Figure 1A, 1B). Similar to SUN1-depleted ALT cells, cell growth was also reduced in SUN1-depleted telomerase-positive cells (Supplementary Figure 1C, 1D). The growth defect caused by SUN1 depletion was previously reported in SUN1-suppressed HeLa1.2.11 cells. SUN1 knockdown can induce robust activation of the checkpoint response and has a dramatic effect on the cell cycle [57]. The dramatic growth defect induced by SUN1 depletion hindered us from observing long-term telomere length alterations in SUN1-depleted cells. However, the short-term depletion of SUN1 did not destroy the integrity of the nuclear lamina (Supplementary Figure 2). These results suggest that SUN1 plays a role in cell growth and the cell cycle.

**Figure 1:**
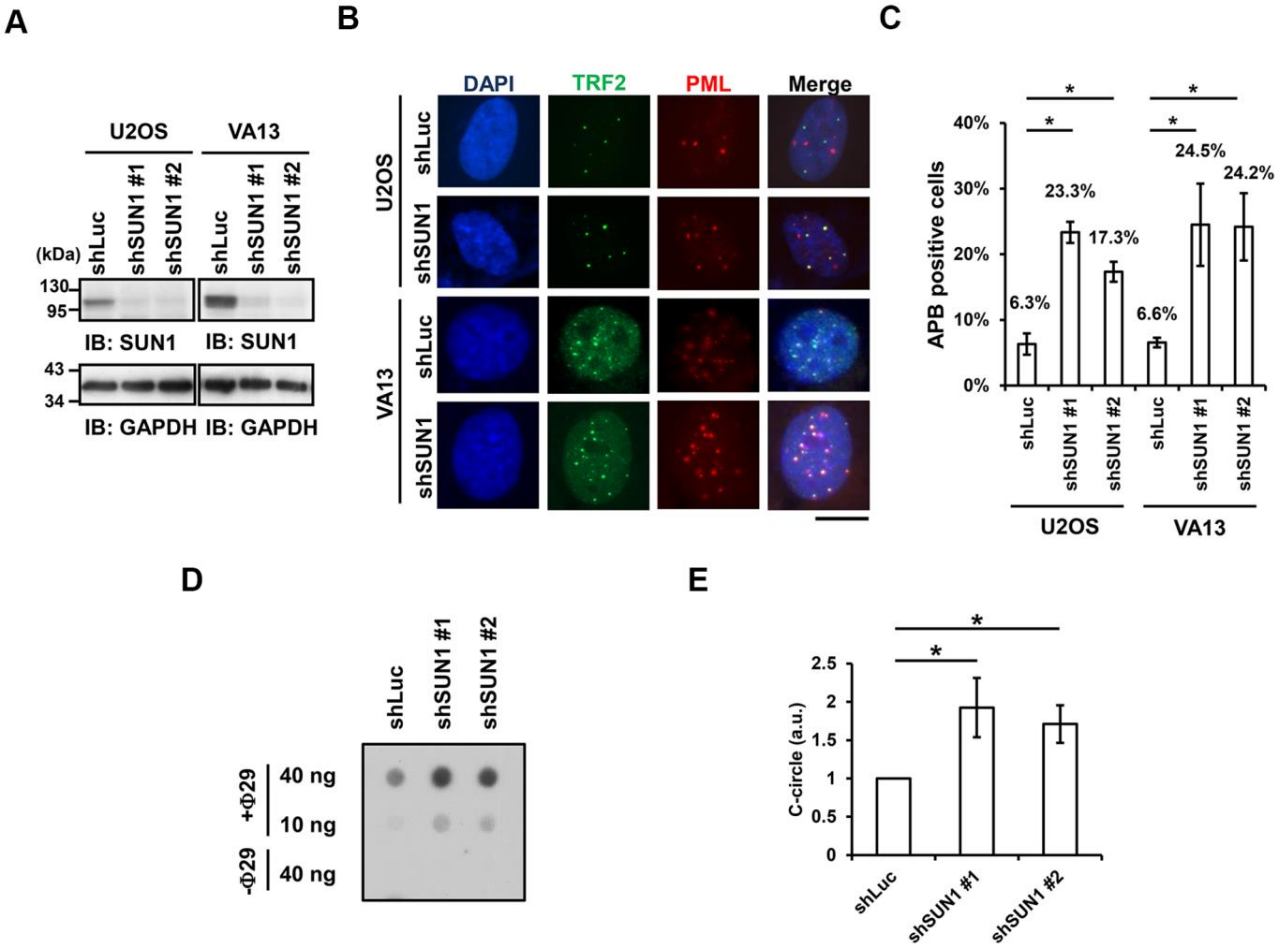
**SUN1 knockdown induces APB formation and C-circle levels.** (**A**) U2OS and VA13 cells were infected with control (shLuc) or shSUN1 lentivirus and selected with 1 μg/ml puromycin for 3 days. Cell lysates were subjected to immunoblot analysis with anti-SUN1 and anti-GAPDH antibodies. GAPDH was used as the loading control. (**B**) Representative images show the colocalization of TRF2 and PML in U2OS cells (upper panel) and VA13 cells (bottom panel). Virus-infected and puromycin-selected cells were subjected to immunofluorescence staining with anti-TRF2 and anti-PML antibodies. DNA was stained with DAPI. Cells containing at least three large TRF2 and PML colocalization foci (yellow) in the nucleus were counted as APB-positive cells. Scale bar, 20 μm. (**C**) Quantification of APBs (%) in the U2OS and VA13 cells shown in (**B**). Approximately 200-300 cells were analyzed for each independent experiment. Error bars denote SD; n=3 (independent experiments); *P<0.05 (two-tailed Student’s t-test). (**D**) Depletion of SUN1 stimulates the formation of C-circles in U2OS cells. (**E**) Quantification of the level of C-circles in the cells in (**D**). The signals were quantified with ImageJ software. The level of C-circles is represented in an arbitrary unit (a.u.). Error bars denote SD; n=3 (independent experiments); *P<0.05 (two-tailed Student’s t-test).

One of the signatures of ALT cells is the formation of APBs, but the mechanism of their formation remains unclear [10, 22]. To understand the function of SUN1 in the ALT pathway, we studied the formation of APBs by examining the colocalization of PML and the telomere-binding protein TRF2. The SUN1 protein levels in the short-term SUN1-depleted U2OS and VA13 ALT cells were confirmed by immunoblotting (Figure 1A). The percentage of the APB-positive cells in the SUN1-depleted ALT cells was significantly increased (Figure 1B, 1C). Under SUN1 depletion, the percentage of the cells exhibiting another ALT cell biomarker, the C-circle [25], was also significantly increased (Figure 1D, 1E). In contrast, no APB formation was observed in the SUN1-depleted telomerase-positive HeLa and HCT116 cells, implying that the loss of SUN1 does not convert telomere-positive cells into telomerase-negative ALT cells (Supplementary Figure 3). These results suggest that SUN1 may play an inhibitory role in the formation of APBs and C-circles.

### The RAP1-SUN1 fusion protein decreases APB formation

**Figure 2:**
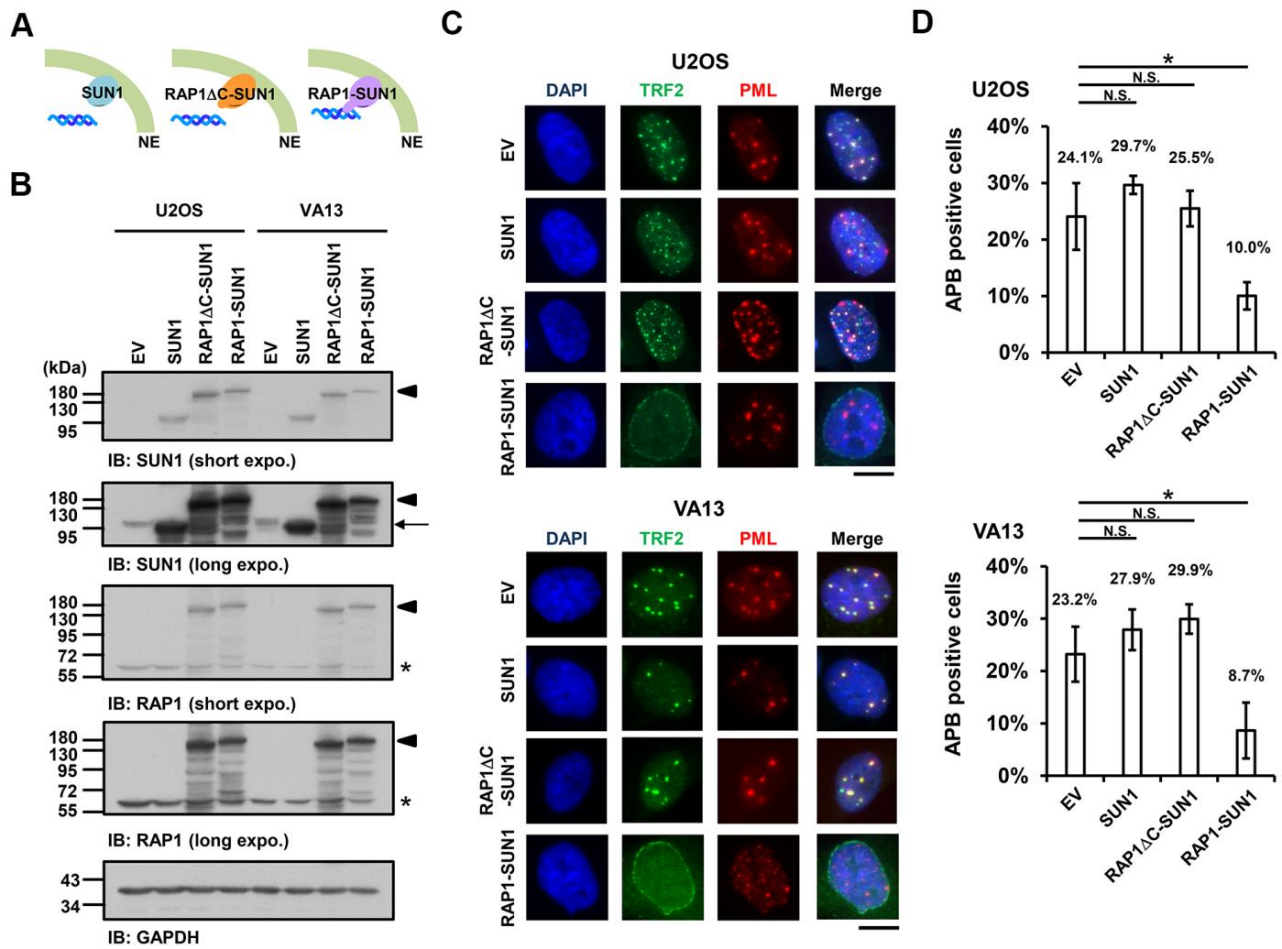
**The enhancement of nuclear envelope anchorage inhibits APB formation.** (**A**) Schematic diagrams of cells overexpressing SUN1, RAP1-RCT-domain-deleted-SUN1 (RAP1ΔC-SUN1), or RAP1-SUN1 fusion chimera protein are shown. NE, the nuclear envelope. (**B**) U2OS and VA13 cells were infected with lentivirus expressing the empty vector control (EV), SUN1, RAP1ΔC-SUN1, or RAP1-SUN1 fusion and then selected in medium containing G418 for 5 days. Cell lysates were analyzed by immunoblotting with anti-RAP1, anti-SUN1, and anti-GAPDH antibodies. The arrowhead indicates the RAP1-SUN1 fusion protein. The arrow indicates endogenous SUN1. The asterisk indicates endogenous RAP1. The ladders under the major protein band show possible products of protein degradation. GAPDH was used as the loading control. (**C**) Representative images show the colocalization of TRF2 and PML in U2OS cells (upper panel) and VA13 cells (bottom panel), as shown in Figure 1. Scale bar, 20 μm. (**D**) Quantification of APBs (%) in the U2OS and VA13 cells shown in (**C**). Approximately 200-300 cells were analyzed for each independent experiment. Error bars denote SD; n=3 (independent experiments); *P<0.05 (two-tailed Student’s t-test). N.S., no significance.

Since the interaction between the nuclear envelope SUN1 protein and telomere shelterin RAP1 protein contributes to the tethering of human telomeres to the nuclear envelope in non-ALT cells [57], we speculated that enhancing telomere-nuclear envelope interactions may decrease APB formation in ALT cells. To test this hypothesis, we generated a RAP1-SUN1 fusion protein (Figure 2A) that connects RAP1 and SUN1 via an eight-glycine linker. The RAP1-SUN1 fusion protein was overexpressed in U2OS and VA13 ALT cells through lentivirus transduction, and the expression levels of the fusion and endogenous proteins were measured with immunoblotting (Figure 2B). It was reported that APBs are usually found in less than 5% of an asynchronous ALT cell population [33]. However, methionine deprivation can arrest cells in the G2 phase and increase the number of APBs in ALT cells [15, 59]. To monitor with efficiency the difference in APB formation between the cells expressing the empty vector and RAP1-SUN1, we depleted methionine in the virus-transduced U2OS and VA13 ALT cells. Overexpression of SUN1 or RAP1 alone did not affect the APB formation (Figure 2C, 2D and Supplementary Figure 4). Coimmunostaining of RAP1 and Lamin A/C demonstrated that the RAP1-SUN1 fusion protein was localized to the nuclear periphery (Supplementary Figure 5A). The DNA-binding protein TRF2 binds to telomeric DNA directly and interacts with RAP1 to form the shelterin complex [58]. Interestingly, some TRF2 proteins localize around the periphery of the nucleus, implying that the function of the RAP1 in the RAP1-SUN1 fusion protein is competent to attract TRF2 to the nuclear envelope (Supplementary Figure 5B). Overexpression of the RAP1-SUN1 fusion protein significantly reduces the APBs formation in both ALT cells (Figure 2C, 2D). Notably, the APB foci were primarily formed in the internal region of the nucleus but rarely in the periphery. Additionally, overexpression of the RAP1-SUN1 fusion protein slowed the cell growth of the ALT- and telomerase-positive cells (Supplementary Figure 6). RAP1 interacts with TRF2 through its RAP1 C-terminal (RCT) protein-protein interaction domain [58]. To examine whether the interaction between RAP1 and TRF2 affects telomere anchorage, we generated the RCT domain-deleted RAP1-SUN1 fusion protein (RAP1ΔC-SUN1) (Figure 2B), which loses the ability of RAP1 to bind to telomeres. As predicted, similar to SUN1 overexpression, RAP1ΔC-SUN1 fusion protein expression did not affect APB formation (Figure 2C, 2D). However, the C-circle levels were not changed in the cells overexpressing RAP1, SUN1, RAP1ΔC-SUN1, or RAP1-SUN1 fusion protein (Supplementary Figure 7). These results indicate that the imposed telomere-nuclear envelope interaction might impede the progression of APB formation.

### SUN1 depletion and RAP1-SUN1 fusion do not affect telomere length during short-term culturing

Because of the growth defect in SUN1-knockdown or RAP1-SUN1-overexpressing cells, we could not acquire long-term cultured ALT cells. We could merely obtain short-term cultured U2OS ALT cells. To evaluate whether telomere length was affected by SUN1 depletion or RAP1-SUN1 overexpression in these short-term-cultured ALT cells, we performed a telomere restriction fragment (TRF) assay. The telomere length in SUN1-depleted cells was not changed after seven days (Supplementary Figure 8A). We next measured the telomere length of SUN1-, RAP1ΔC-SUN1-, and RAP1-SUN1-overexpressing cells. The telomere lengths in these cells did not show significant variation compared to those in the control cells (Supplementary Figure 8B). These results reveal that telomere length homeostasis is not changed in SUN1-depleted or RAP1-SUN1-overexpressed short-term cultures.

### Depletion of SUN1 alleviates the requirement of TOP3α in ALT cell

**Figure 3:**
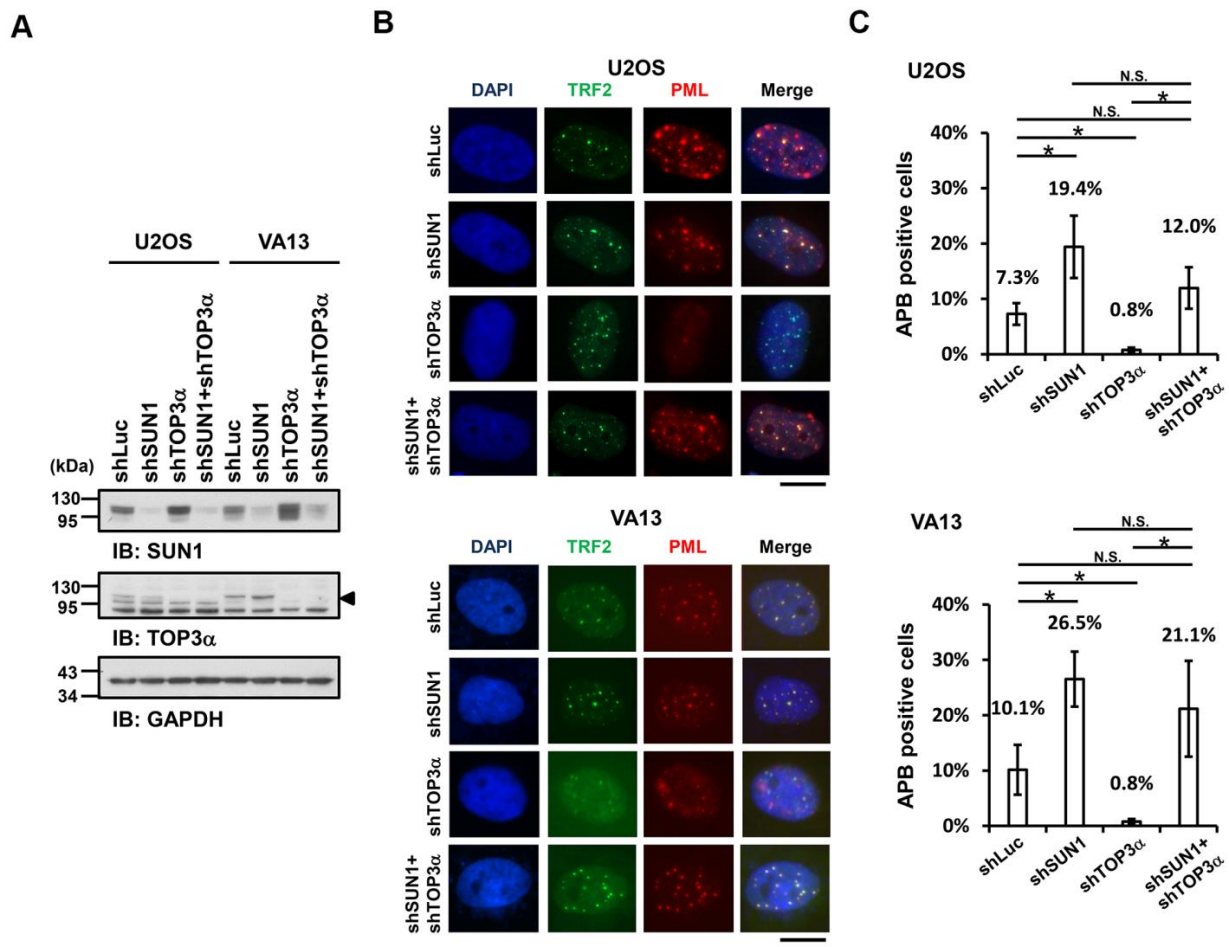
**SUN1 depletion increases APB formation in TOP3α-knockdown cells.** (**A**) U2OS and VA13 cells were infected with control (shLuc), shSUN1, shTOP3α, or shSUN1 combined with shTOP3α lentiviruses and selected for 3 days. Cell lysates were analyzed by immunoblotting with anti-SUN1, anti-TOP3α, and anti-GAPDH antibodies. The arrowhead indicates the location of the TOP3α protein. GAPDH was used as the loading control. (**B**) Representative images show the colocalization of TRF2 and PML in U2OS cells (upper panel) and VA13 cells (bottom panel), as shown in Figure 1. Scale bar, 20 μm. (**C**) Quantification of APBs (%) in the U2OS and VA13 cells shown in (**B**). Approximately 200-300 cells were analyzed for each independent experiment. Error bars denote SD; n=3 (independent experiments); *P<0.05 (two-tailed Student’s t-test). N.S., no significance.

Previous studies demonstrated that telomere-telomere recombination specifically requires TOP3α to resolve highly negative topological stress generated during fork movement along telomeres undergoing recombination [60, 61]. If the supercoiling stress is at least partly derived from the anchorage of telomeres to the nuclear envelope, detaching the telomeres from the nuclear envelope may allow free rotation of the telomeres during recombination and thereby relieve the supercoiling tension. To examine this possibility, we depleted TOP3α in ALT cells to evaluate the APB formation ability (Figure 3A). The cell proliferation rates were also analyzed (Supplementary Figure 9). As predicted, depletion of TOP3α abolished APB formation, while depletion of SUN1 in the TOP3α-depleted cells led to recovered APB formation (Figure 3B, 3C). These results may imply that the detachment of telomeres from the nuclear envelope alleviates the requirement of TOP3α to resolve topological stress.

### Coil domain phosphorylation-mimetic mutations of RAP1 block the RAP1-SUN1 interaction

**Figure 4:**
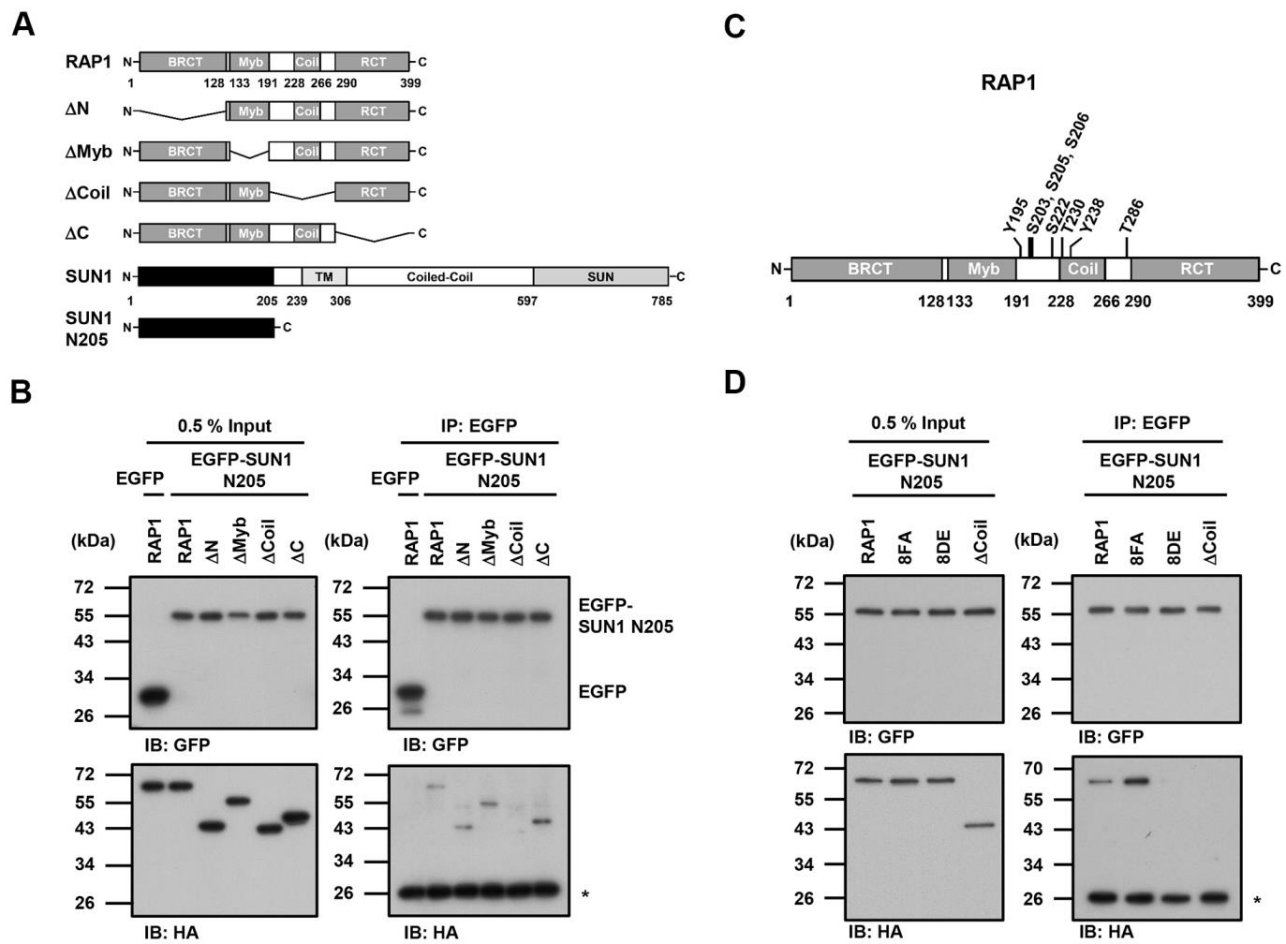
**The coil region of RAP1 and the potential phosphorylation of residues in that domain are likely critical for the SUN1 interaction.** (**A**) Schematic representations of the N-terminal HA-tagged RAP1 constructs and the N-terminal EGFP-tagged SUN1 constructs. TM, transmembrane. (**B**) U2OS cells were transfected with HA-tagged RAP1 together with either EGFP or EGFP-tagged SUN1 N205. Forty-eight hours posttransfection, the cells were harvested for use in immunoprecipitation assays. EGFP-SUN1 N205 was immunoprecipitated with anti-GFP beads. Input and immunoprecipitated proteins (IPs) were analyzed by immunoblotting with anti-GFP and anti-HA antibodies. Asterisk (*), nonspecific band. (**C**) A schematic representation of the eight potential phosphorylation sites in the coil domain of RAP1. (**D**) U2OS cells were transfected with HA-tagged RAP1 WT, nonphosphorylatable (8FA), or phospho-mimetic (8DE) mutant together with EGFP-tagged SUN1 N205. Forty-eight hours posttransfection, the cells were harvested for use in immunoprecipitation assays. Input and immunoprecipitated proteins (IPs) were analyzed by immunoblotting with anti-GFP and anti-HA antibodies. Asterisk (*), nonspecific band.

Since the detailed mechanism of the RAP1-SUN1 interaction is not fully understood, to determine which RAP1 domain interacts with SUN1, we generated various truncated forms of RAP1 for domain mapping (Figure 4A). The N-terminal domain of SUN1 is located in the nucleoplasm and interacts with RAP1, while the SUN1 C-terminus is located in the perinuclear space that anchors the protein to the nuclear envelope [47, 57]. Due to the difficulty of precipitating insoluble membrane proteins, we constructed a truncated form of SUN1 that contains 205 N-terminal soluble amino acids (SUN1 N205), which preserves the ability of SUN1 to interact with RAP1. U2OS cells were cotransfected with an HA-tagged RAP1 plasmid together with the EGFP-tagged SUN1 N205 plasmid to perform immunoprecipitation. Full-length HA-tagged RAP1 was efficiently coimmunoprecipitated with EGFP-tagged SUN1 N205 but not EGFP alone (Figure 4B). However, coil-deleted (ΔCoil) RAP1 was not coprecipitated with SUN1 (Figure 4B), suggesting that the coil region of RAP1 is critical for SUN1 binding.

Many protein-protein interactions are modulated through posttranslational modifications, such as phosphorylation. We were curious to know whether the interaction between human RAP1 and SUN1 may be regulated by RAP1 phosphorylation. We analyzed the potential phosphorylation of RAP1 using the proteomic PhosphoSitePlus^®^ database (https://www.phosphosite.org/homeAction.action). There are 22 phosphorylation sites on RAP1, and eight phosphorylation sites are located within the coil region (Figure 4C). To characterize the kinases of these eight phosphorylation sites, the KinasePhos 2.0 website was used to predict the potential kinases (Table 1) [62]. Based on the analysis, S203, S205, S206, and S222 might be regulated by ATM kinase, Y195 and Y238 might be regulated by FGFR1, and T286 might be regulated by CK2. Next, we replaced these eight tyrosine, serine, or threonine residues with nonphosphorylatable phenylalanine and alanine (8FA) and phospho-mimetic aspartic acid and glutamic acid (8DE). Interestingly, the nonphosphorylatable RAP1-8FA mutations showed enhanced interactions with SUN1, but phospho-mimetic RAP1-8DE mutations lost the ability to interact with SUN1 (Figure 4D). These results suggest that this potential phosphorylation in the coil region of RAP1 might inhibit the interaction between RAP1 and SUN1.

**Table 1:** Putative phosphorylation sites of RAP1 coil domain.

**Phosphorylation site**	**Sequence (N’→C’)**	**Predicted kinase^a^**	**Score^a^**	**References**
Y195	192 EHKYLLG	FGFR1	0.53	
S203	220 APVSPSS	ATM	0.93	[87]
S205	202 VSPSSQK	ATM	0.97	[85]
S206	203 SPSSQKL	ATM	0.93	
S222	219 AADSGEP	ATM	0.99	
T230	227 NKRTPDL	GRK	0.54	
Y238	235 EEEYVKE	FGFR1	0.53	
T286	283 DPPTPEE	CK2	0.59	

^a^Kinases were predicted and scored by KinasePhos2.0 (http://kinasephos2.mbc.nctu.edu.tw/index.html) [62].

### RAP1-SUN1 interaction-independent nuclear envelope tethering pathways participate in APB formation

**Figure 5:**
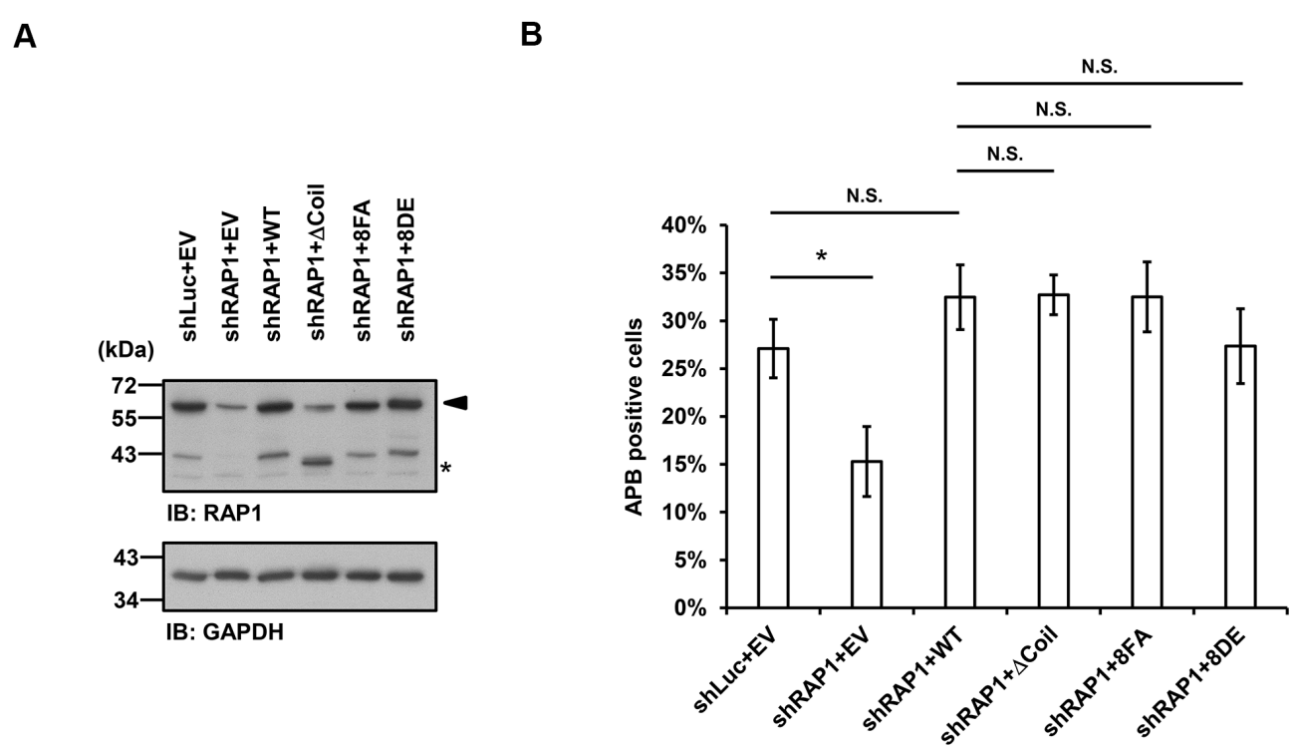
**Disruption of the interaction between RAP1 and SUN1 does not interfere with APB formation in ALT cells.** (**A**) U2OS cells were infected with the knockdown control (shLuc) or shRAP1 lentivirus and simultaneously complemented with control (EV), wild-type RAP1 (WT), RAP1 coil deletion (ΔCoil), nonphosphorylatable (8FA), or phospho-mimetic (8DE) RAP1 mutant lentiviruses. Virus-infected cells were selected for 5 days and subjected to further methionine restriction for 3 days. Cell lysates were analyzed by immunoblotting with anti-RAP1 and anti-GAPDH antibodies. The arrowhead indicates the location of endogenous RAP1, and the multiple lower-molecular-weight bands are degraded RAP1. Asterisk (*), RAP1 coil deletion mutant. GAPDH was used as the loading control. (**B**) Quantification of APBs (%) in the U2OS cells shown in (**A**). Approximately 200-300 cells were analyzed for each independent experiment. Error bars denote SD; n=3 (independent experiments); *P<0.05 (two-tailed Student’s t-test). N.S., no significance.

Finally, we investigated whether APB formation may be affected by the observed RAP1-SUN1 interaction. We simultaneously introduced RAP1-knockdown lentiviruses, which targeted the 3’ UTR of RAP1, and knockdown-resistant RAP1-expressing lentiviruses into U2OS cells. We also deprived methionine in the medium to enrich the APB-positive subpopulations. The protein expression levels were determined by immunoblotting (Figure 5A). It has been reported that the depletion of RAP1 hampers APB formation [33]. Consistent with this previous report, RAP1 knockdown in the U2OS cells led to decreased APB formation (Figure 5B). However, enforced expression of wild-type RAP1 to the endogenous level restored APB formation in the RAP1-depleted cells (Figure 5A, 5B). In addition, the cells expressing the RAP1 coil deletion and the nonphosphorylatable RAP1-8FA and phospho-mimetic RAP1-8DE mutants all displayed the same level of APBs, which was similar to that in the cells expressing wild-type RAP1 (Figure 5B). Our data imply that a SUN1-dependent but RAP1-independent pathway might contribute to APB formation in ALT cells.

## DISCUSSION

**Figure 6:**
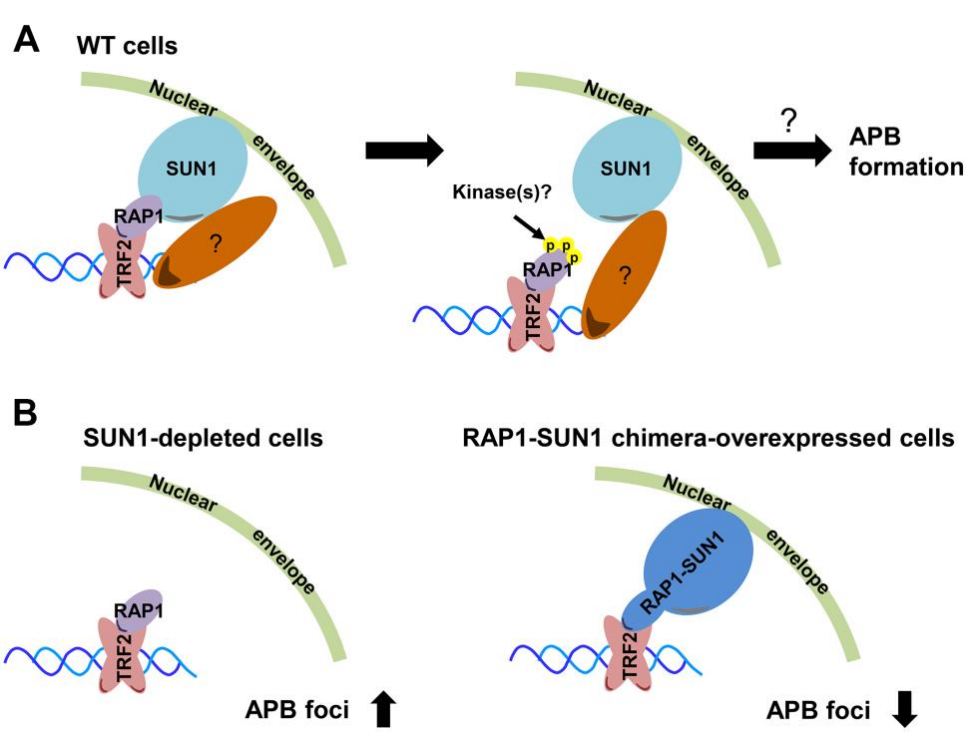
**Model depicting the role of RAP1-SUN1-mediated telomere-nuclear envelope attachment during telomere-telomere recombination in ALT cells.** (**A**) The interaction between RAP1 and SUN1 contributes to telomere anchorage to the nuclear envelope. Unknown kinases might phosphorylate the coil domain of RAP1, inducing RAP1 release from SUN1. Additionally, SUN1 might connect with an unknown telomere-binding protein, and this interaction may provide another telomere-nuclear envelope tethering mechanism to constrain the telomere from freely roaming. The molecular mechanism of how the telomeres depart from the nuclear envelope to the APB remains a mystery. (**B**) The depletion of SUN1 leads to the release telomeres from the nuclear envelope anchorage and increases the APB formation. However, the RAP1-SUN1 chimera enforces anchorage and decreases the APB formation in ALT cells.

The first cytological evidence for telomere anchoring to the nuclear membrane was discovered by the Gasser laboratory [63]. In budding yeast, telomeres are clustered and placed at the nuclear periphery, where they create a nuclear subcompartment for telomere silencing [64]. It was reported that yeast subtelomeric Y’ recombination is suppressed by telomere anchorage to the nuclear envelope [65]. Moreover, the yeast telomere-binding protein Rif1 is required for telomere anchorage [66]. In human telomerase-positive cells, telomere-nuclear envelope anchorage occurs during the cell cycle and is at least partly mediated through the interaction between the shelterin subunit RAP1 and the nuclear envelope protein SUN1 [57]. Previous studies have also shown that in certain non-ALT cells, most telomeres are located within the nuclear interior during interphase [40, 67]. However, the telomere-nuclear envelope association in ALT cells had not been explored. Here, we show that SUN1 might play a role in preventing telomere-telomere recombination. We also reveal that potential phosphorylation at the RAP1 coil domain might decrease the interaction between RAP1 and SUN1. We hypothesize that with unknown environmental or internal stimuli, some unidentified kinases may phosphorylate the RAP1 coil domain to induce the dissolution of the RAP1 and SUN1 interaction. The detailed mechanism of how telomeres leave the nuclear envelope and then generate APBs remains unclear (Figure 6A).

We observed that the APB formation is increased in SUN1-depleted ALT cells but is decreased in RAP1-SUN1 fusion ALT cells (Figure 6B). In budding and fission yeasts, SUN1 homologs sequester double-strand breaks (DSBs) at the nuclear envelope to protect them from deleterious recombination [42, 43]. Based on these findings, one possible mechanism of SUN1-suppressed APB formation might be based on SUN1 sequestering telomeres to prevent their homologous recombination. Another plausible mechanism is that the SUN1-RAP1 interaction may generate supercoiling tension that impedes subsequent telomere-telomere recombination. Crabbe and her colleagues revealed that not all telomeres are located in the nuclear interior during the cell cycle in telomerase-positive cells. In S phase, 25% of telomeres are located around the nuclear envelope. Furthermore, when entering the subsequent G1 stage, these cells retain approximately 40% of telomeres at the nuclear envelope [57]. We proposed that in SUN1-depleted ALT cells, the tethering force of telomeres on the nuclear envelope may partially loosen, which might provide more telomere flexibility and favor APB formation. In contrast, in RAP1-SUN1-expressing ALT cells, telomeres are forced “locked” with the nuclear envelope, which might enhance the difficulty of executing telomere-telomere recombination. Furthermore. ALT cells require TOP3α to resolve topological stress caused by telomere-telomere recombination [60, 61]. We also found that knocking down SUN1 in TOP3α-depleted ALT cells restored APB formation, which is consistent with the hypothesis that the topoisomerase activity of TOP3α is dispensable in ALT cells when some telomeres are not tethered to the nucleus.

Telomere extension in ALT cells is mainly generated by the break-induced DNA replication (BIR) pathway [68–72]. Recently, it was discovered that ALT cells harbor bifurcated RAD52-dependent and RAD52-independent BIR pathways that lead to elongated telomeres. Both BIR pathways are involved in APB formations during ALT cell DNA synthesis, but only the RAD52-independent BIR pathway is critical for C-circle formation [18]. Telomeric DNA break-induced replication fork collapse and telomere damage-induced internal loops are both involved in C-circle formation [73, 74]. In SUN1-depleted cells, both the APB formation and C-circle levels were increased. Nevertheless, overexpression of the RAP1-SUN1 fusion protein constrains only APB formation but not C-circle levels. We speculate that ALT telomeric DNA might be more fragile to DNA replication fork-induced topological stress when the telomere ends are locked to the nuclear envelope. Subsequently, DNA replication fork collapse or DNA breakage might occur more frequently to induce C-circle formation.

Budding yeast contains two telomere-nuclear envelope anchoring pathways at different cell cycle stages. The telomere-associated proteins Sir4 and yKu contact the nuclear inner membrane proteins Mps3 and Esc1, respectively, at different cell cycle stages [75]. In mammals, while telomeres can be tethered to the nuclear envelope via the RAP1-SUN1 interaction, cells with RAP1 depletion still retain approximately 40% of telomeres at the nuclear periphery after mitosis [57]. Here, we found that RAP1 interacts with SUN1 in the nucleoplasm via its coil region, and the phospho-mimetic mutant impedes the RAP1-SUN1 interaction. However, complementing RAP1-depleted cells with the RAP1 coil deletion or phosphoryl-mimetic mutant did not increase the APB formation. Considering this discrepancy, we hypothesize that another nuclear tethering force might influence APB formation. SUN1 might simultaneously bind another telomeric protein (Figure 6A). Another possibility is that another tethering mechanism bridges telomeres to the nuclear envelope. These possibilities might explain why we could not specifically detect the contribution of the RAP1-SUN1 interaction to APB formation. Cho and his colleagues found that homology searching and directional telomere movement in ALT cells is based on a meiosis-specific complex to perform nonsister homologous chromatid repair [76]. Moreover, in the mammalian meiotic cell cycle, a RAP1-independent telomere-nuclear envelope tethering pathway indeed exists [77]. The nuclear envelope protein complex TERB1/2-MAJIN provides alternative telomere-nuclear membrane attachment during meiosis, suggesting that the telomere-TERB1-TERB2-MAJIN-nuclear envelope associating with the telomere-LINC complex may cooperatively recruit telomeres to the nuclear envelope during meiosis [78, 79]. Recent studies have shown that SUN1 interacts with MAJIN and TERB1 [80, 81]. Therefore, a possible explanation is that ALT cells might borrow meiotic proteins, such as the TERB1/2-MAJIN complex, to orchestrate telomere attachment during the mitotic cell cycle.

In the fission yeast *Schizosaccharomyces pombe*, telomeres are tethered to the nuclear envelope in interphase via the interaction between Rap1 (a homolog of human RAP1) and the inner nuclear membrane protein Bqt4, which connects telomeres to the nuclear envelope during both vegetative growth and meiosis [82]. The dissociation of Rap1 from Btq4 is promoted by CDK-mediated Rap1 hyperphosphorylation at the early M phase, which is required for faithful chromosomal segregation [83]. In fact, in contrast to the “closed mitosis” of *S. pombe*, higher eukaryotes usually undergo nuclear envelope breakdown to release their chromosomes from the nuclear envelope [5]. Although human RAP1 phosphorylation may repress the RAP1-SUN1 interaction, the molecular details of this process in ALT cells remain to be elucidated in further investigations.

This study revealed that telomere-nuclear envelope anchorage interferes with the telomere-telomere recombination pathway in ALT cells. Incomplete telomere detachment from the nuclear envelope may cause the dysregulation of chromatin dynamics. As cell cycle checkpoint pathways, the maintenance of the homeostasis between the nuclear envelope and telomeres may be tightly regulated and important for cell cycle progression. Interestingly, a recent study showed that the mislocalization of LINC complex proteins is a significant characteristic of cellular senescence [84]. Notably, RAP1 S205 phosphorylation, which is activated by p90RS activation, induces the nuclear export of the RAP1-TRF2 complex and further contributes to senescence and telomere dysfunction in epithelial cells [85, 86]. When telomeres are shortened, the ALT pathway is induced by complicated DNA damage signaling and (epi)genetic modification aberrations [22]. Therefore, the correlation between the LINC complex and RAP1 phosphorylation in the progression of senescence and how homeostasis between telomeres and the nuclear envelope is maintained will be important issues for further determination.

## Supplementary Material

Supplementary Figures

Supplementary Tables
