# Supplementary material for: Nuclear envelope tethering inhibits the formation of ALT-associated PML bodies in ALT cells
Source: Aging (Albany NY). 2021 Apr 4;13(7):10490–516. doi: 10.18632/aging.202810 (PMC8064153; doi:10.18632/aging.202810)
Supplement: Supplementary Figures [file aging-13-202810-s001.pdf]

SUPPLEMENTARY FIGURES

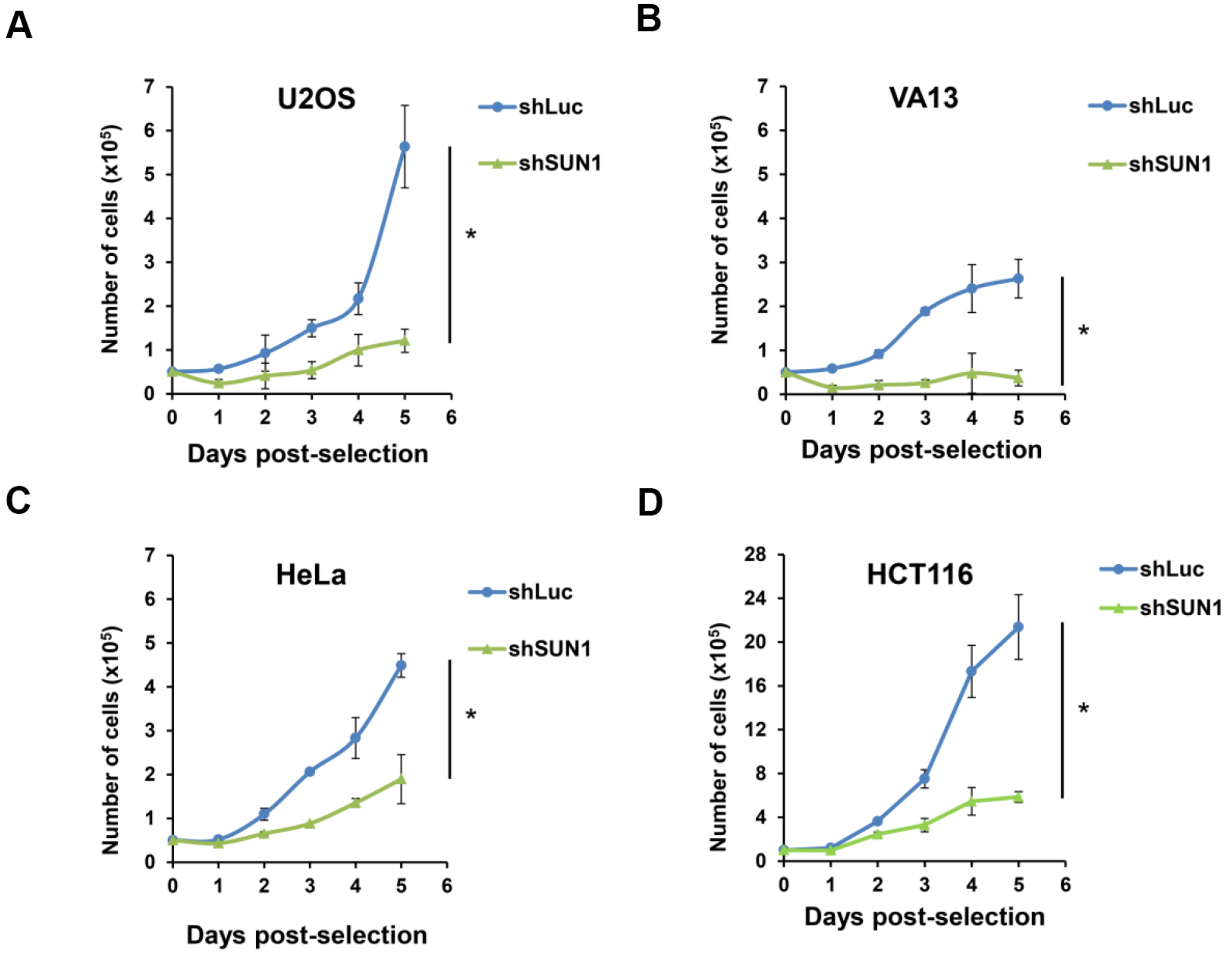

Supplementary Figure 1. Depletion of SUN1 reduces cell growth. The growth curves of the control (shLuc) and knockdown of SUN1 (shSUN1) in two telomerase-negative ALT cell lines, (**A**) U2OS cells and (**B**) VA13 cells, and two telomerase-positive cell lines, (**C**) HeLa cells and (**D**) HCT116 cells, are shown. After lentivirus infection, the cells were selected with puromycin. Cell numbers were counted during drug selection. Error bars denote SD; n=3 (independent experiments); **P*<0.05 (two-tailed Student's t-test).

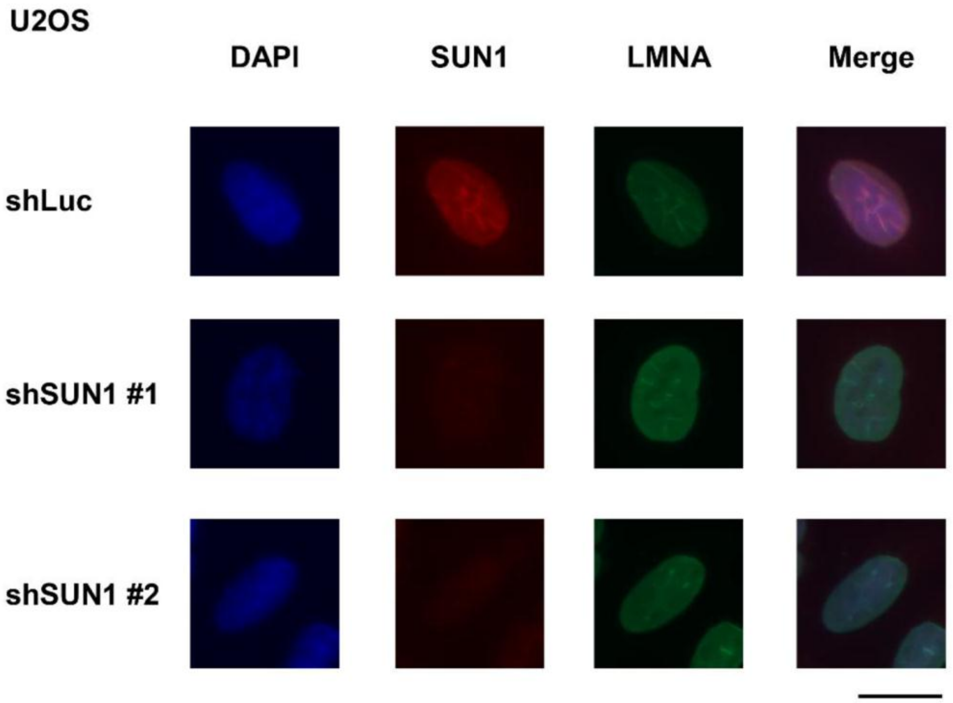

Supplementary Figure 2. Cells with SUN1 depleted maintain the intact structure of the nuclear lamina. U2OS cells were infected with control (shLuc) or shSUN1 lentiviruses and selected with puromycin for 3 days. The cells were used to perform immunofluorescence assays with anti-SUN1 (EPR6554, ab124770, Abcam) and anti-Lamin A/C (N-18, sc-6215) antibodies. DNA was stained with DAPI. Scale bar, 20 µm.

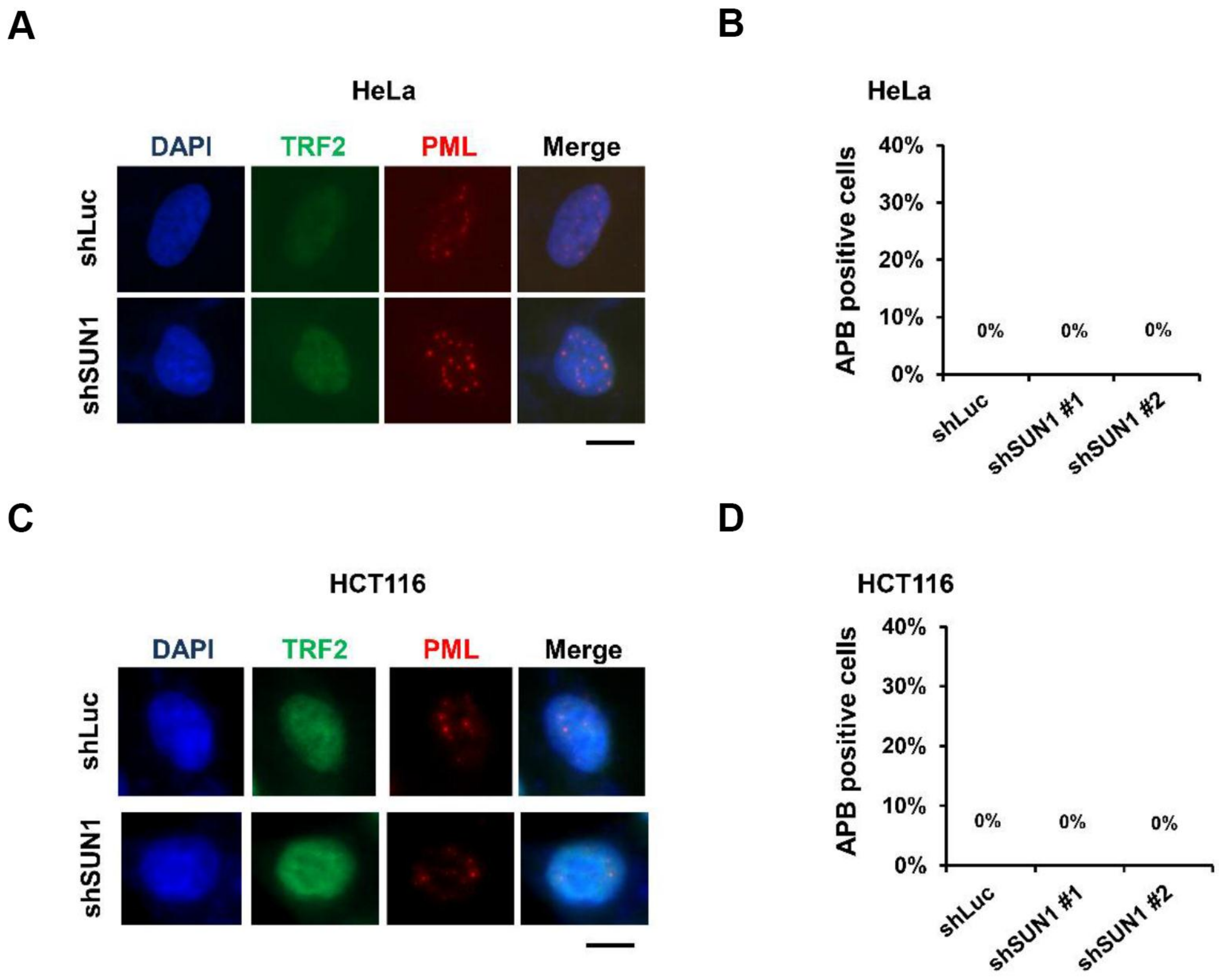

Supplementary Figure 3. Depletion of SUN1 does not induce APB formation in telomerase-positive cells. Telomerase-positive HeLa and HCT116 cells were infected with control (shLuc) or SUN1 knockdown (shSUN1) lentiviruses and selected with puromycin for 3 days. Representative images show that no APB foci were formed in the SUN1-depleted (**A**) HeLa cells or (**C**) HCT116 cells. Quantifications of APBs (%) in the (**B**) HeLa cells and (**D**) HCT116 cells were shown, respectively. Approximately 200-300 cells were analyzed for each independent experiment. Error bars denote SD; n=3 (independent experiments). Scale bar, 20 µm.

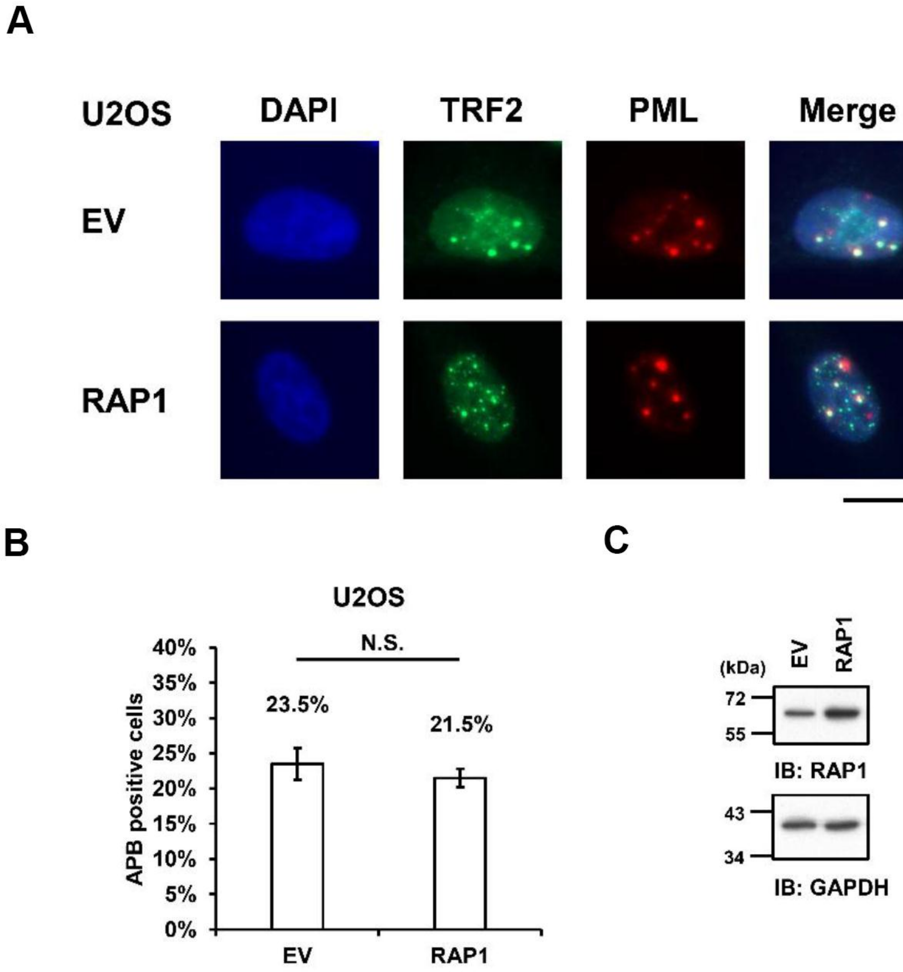

Supplementary Figure 4. Overexpression of the RAP1 protein alone does not significantly reduce the APB formation. (**A**) Representative images show the APBs formed in empty vector control (EV)-expressing or RAP1-expressing U2OS cells. Immunostaining was performed as described in Figure 1. Scale bar, 20 μm. (**B**) Quantification of APBs (%) in the U2OS cells shown in (**A**). Approximately 200-300 cells were analyzed for each independent experiment. Error bars denote SD; n=3 (independent experiments); N.S., no significance (two-tailed Student's t-test). (**C**) Cell lysates were immunoblotted with anti-RAP1 and anti-GAPDH antibodies. GAPDH was used as the loading control.

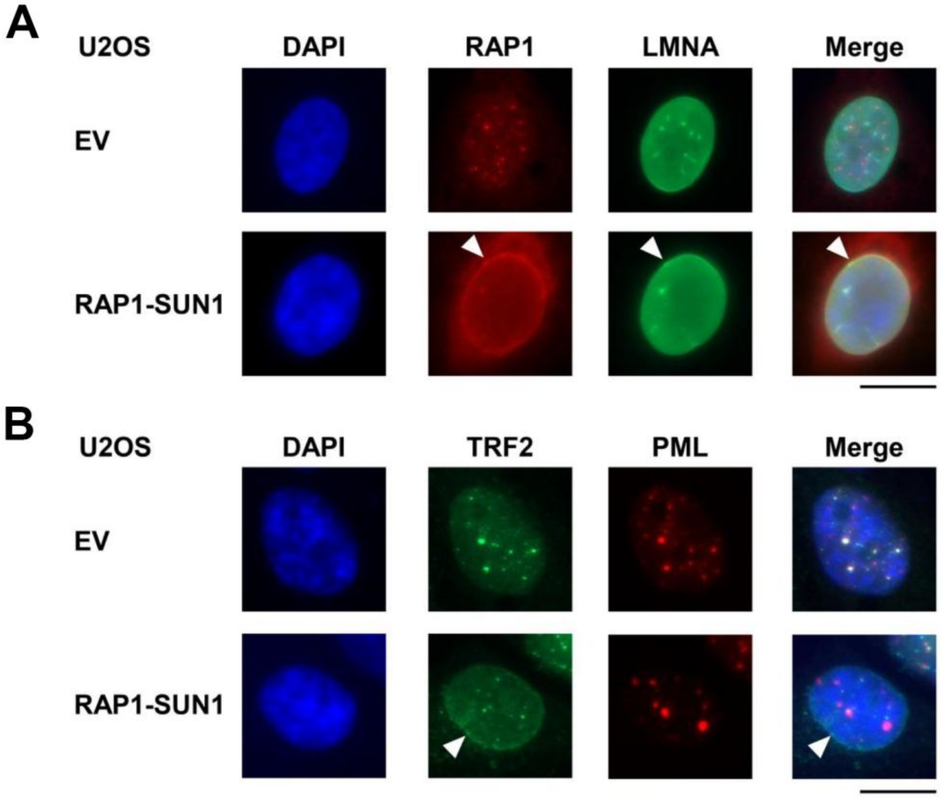

Supplementary Figure 5. RAP1-SUN1 fusion proteins are located around the periphery of the nucleus and competent to recruit TRF2. U2OS cells were infected with lentivirus expressing the empty vector control (EV) or RAP1-SUN1 fusion protein and then selected in medium containing G418- for 5 days. (**A**) Representative images show the colocalization of RAP1-SUN1 and Lamin A/C in U2OS cells. The arrowhead indicates the colocalization site. (**B**) Representative images show that TRF2 appears around the nuclear periphery in the RAP1-SUN1-overexpressing U2OS cells (arrowhead). Scale bar, 20 µm.

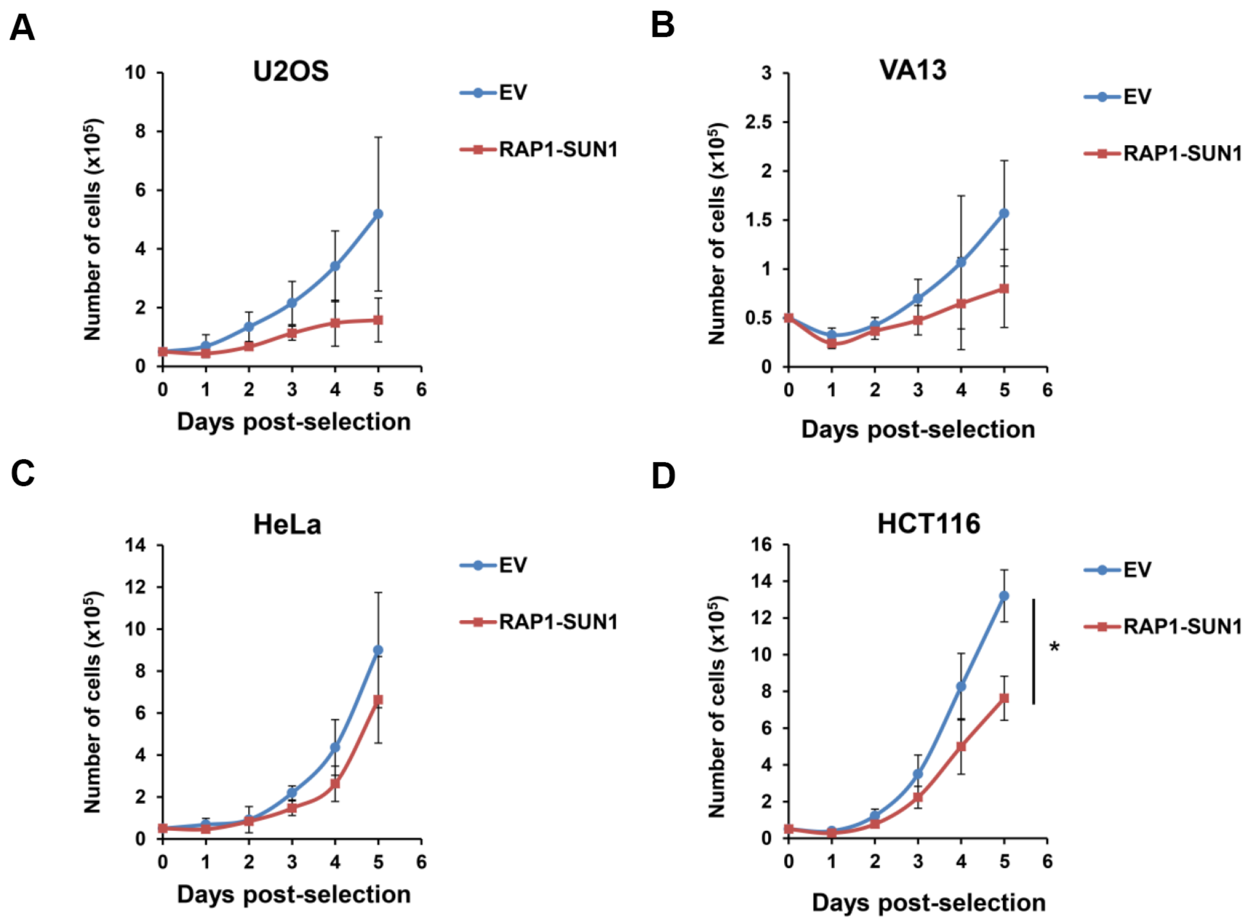

Supplementary Figure 6. Expression of the RAP1-SUN1 fusion protein reduces cell growth. The growth curves of cells expressing the empty vector (EV) or RAP1-SUN1 fusion protein (RAP1-SUN1) in two telomerase-negative ALT cell lines, (**A**) U2OS cells and (**B**) VA13 cells, and two telomerase-positive cell lines, (**C**) HeLa cells and (**D**) HCT116 cells, are shown. After lentivirus infection, cells were the selected for G418. Cell numbers were counted during drug selection. Error bars denote SD; n=3 (independent experiments); * $P<0.05$ (two-tailed Student's t-test).

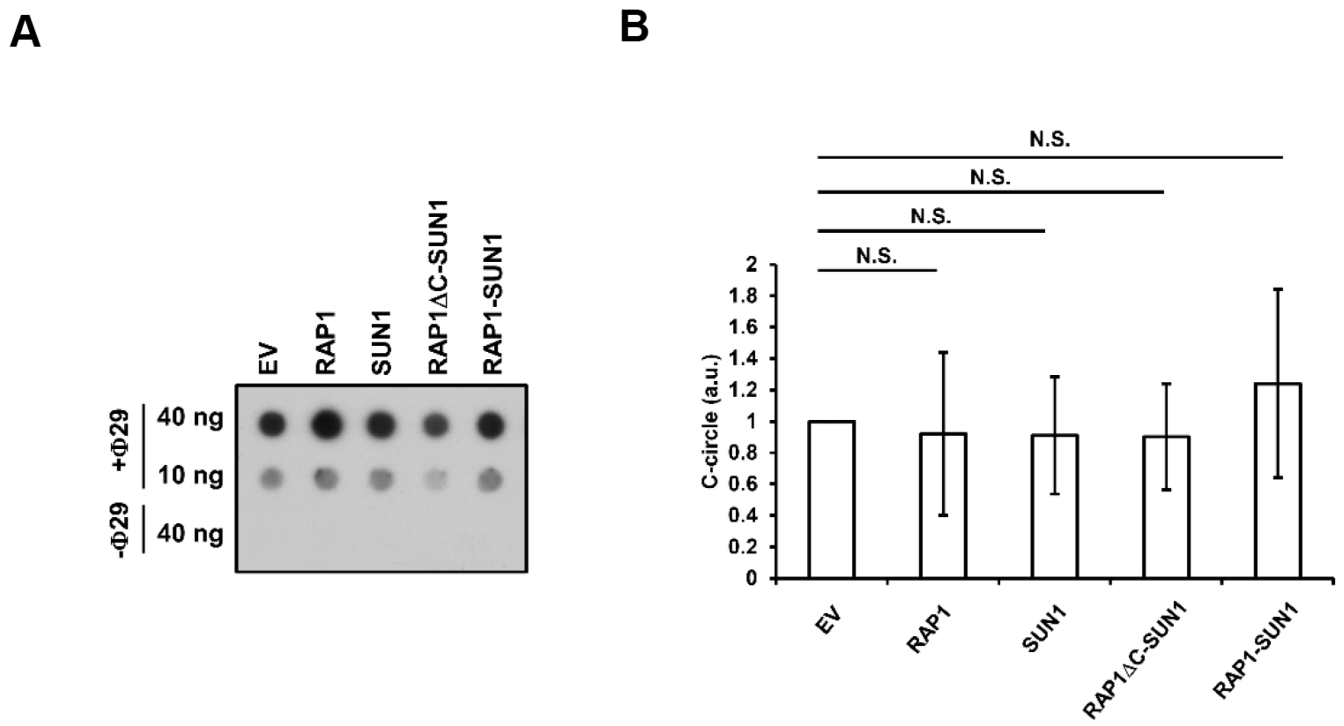

Supplementary Figure 7. Overexpression of the RAP1-SUN1 fusion protein did not significantly change the C-circle level in U2OS cells. (**A**) The C-circle level in U2OS cells expressing the empty vector (EV), RAP1, SUN1, RAP1ΔC-SUN1, or RAP1-SUN1 fusion protein. (**B**) Quantification of the level of C-circles shown in (**A**). The C-circle signals were quantified with ImageJ software. The level of C-circles is represented in an arbitrary unit (a.u.). Error bars denote SD; n=3 (independent experiments); N.S., no significance (two-tailed Student's t-test).

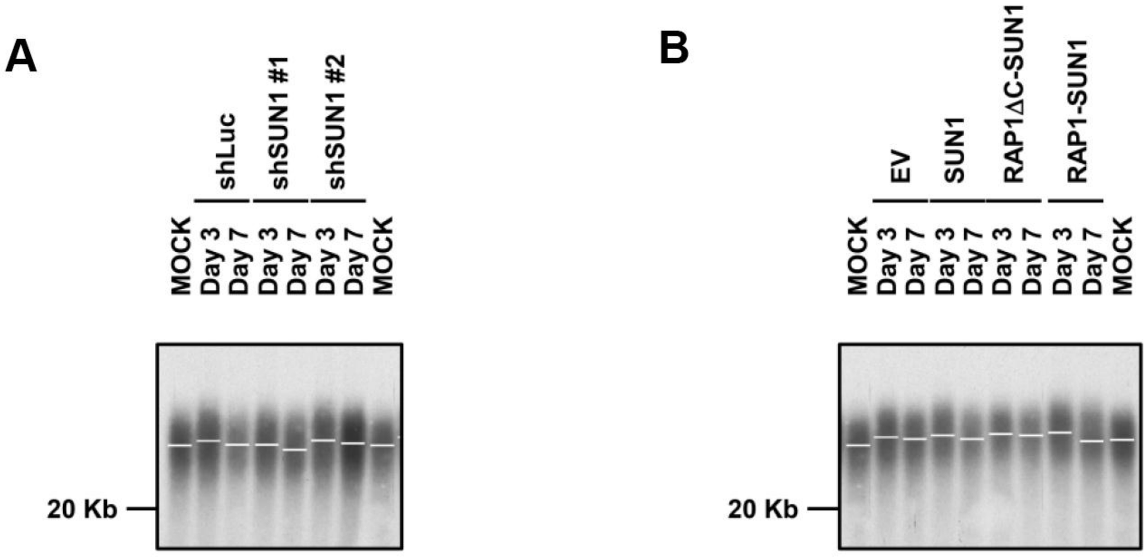

Supplementary Figure 8. Knockdown or overexpression of SUN1 does not disturb the homeostasis of telomere length in cells in short-term culture. (**A**) U2OS cells were infected with control (shLuc) or SUN1-knockdown (shSUN1) lentiviruses. The cells were selected with 1 μg/ml puromycin for 3 days and 7 days, respectively. Telomere length was measured by TRF assay. The peak intensity of telomere length is indicated by a white line. (**B**) U2OS cells were infected with lentiviruses overexpressing the control (EV), SUN1, RAP1ΔC-SUN1 fusion, or RAP1-SUN1 fusion protein. The cells were selected, and telomere length was measured as described in (**A**).

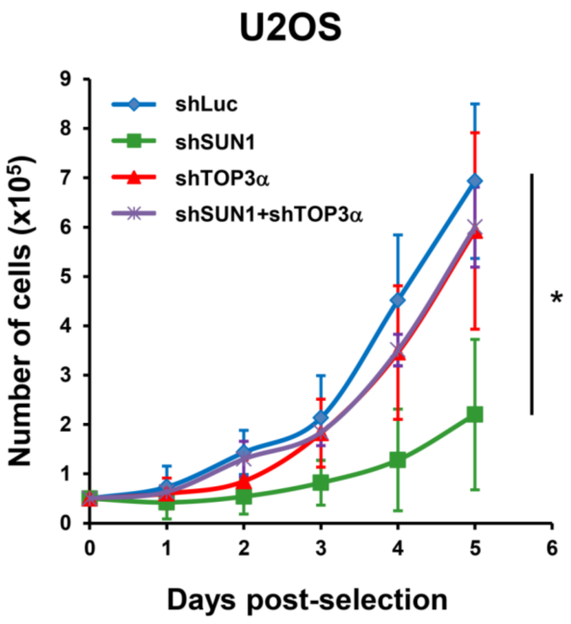

Supplementary Figure 9. Growth curves of the shLuc, shSUN1, shTOP3α, and shSUN1+shTOP3α ALT cells. The growth curves of U2OS cells within 5 days postselection are shown. Error bars denote SD; n=3 (independent experiments); * $P<0.05$ (two-tailed Student's t-test).
