# Supplementary material for: Nuclear envelope tethering inhibits the formation of ALT-associated PML bodies in ALT cells
Source: Aging (Albany NY). 2021 Apr 4;13(7):10490–516. doi: 10.18632/aging.202810 (PMC8064153; doi:10.18632/aging.202810)
Supplement: Supplementary Tables [file aging-13-202810-s002.pdf]

SUPPLEMENTARY TABLES

Supplementary Table 1. Oligo sequences for shRNA-mediated gene knockdown.

Clone ID	Gene symbol	Target sequence	Region
TRCN0000279614	*SUN1*	GAACTAGAACAGACCAAGCAA	CDS
TRCN0000133655	*SUN1*	GCTGTTCTGAAACTTACGAAA	CDS
TRCN0000049297	*TOP3A*	GCTTCTCGAAAGTTGAGAATA	CDS
TRCN0000295900	*TERF2IP* (*RAP1*)	GAGAGTTCTTGCATTGGAACT	3'UTR

Supplementary Table 2. Primers used in this study.

Primer	Sequence
	pLAS5w-RAP1
RAP1-NheI-For	CCACGTGGCGATCGCTAGCGCCACCATGGCGGAGGCGATGGATTTGGGCAAAGACCCCAA
RAP1-NsiI-Rev	CTGTACATGCATTTATTTCTTTCTAAATTCAATCCTCCGAGCTACATTCTGAGCACC
	pLAS5w-SUN1
NheI-SUN1-For	GCTAGCATGGATTTTTCTCGGCTTCA
SUN1-EcoRI-Rev	GAATTCTCACTTGACAGGTTCGCCAT
	pLAS5w-RAP1-SUN1
HpaI-RAP1-For	GTTAACATGGCGGAGGCGATGGATTT
RAP1-G8-NheI-Rev	GCTAGCACCACCACCACCACCACCACCACCTTTCTTTCGAAATTCAATCC
NheI-SUN1-For	GCTAGCATGGATTTTTCTCGGCTTCA
SUN1-EcoRI-Rev	GAATTCTCACTTGACAGGTTCGCCAT
	pLAS5w-RAP1 ΔC-SUN1
HpaI-RAP1-For	GTTAACATGGCGGAGGCGATGGATTT
RCTdel-G8-NheI-Rev	GCTAGCACCACCACCACCACCACCACCACC TGAGTCTTCCTCAGGTGTGGGTGGATCATC
	pcDNA3HA-RAP1
RAP1-BamHI-1F	GGATCCAATGGCGGAGGCGATGGATTTG
RAP1-XhoI-R	CTCGAGTTATTTCTTTCGAAATTC
	pcDNA3HA-RAP1 ΔN
hRAP1-BamH1-129F	GGATCCACGGCACGCCGGGCGGATC
RAP1-XhoI-R	CTCGAGTTATTTCTTTCGAAATTC
	pcDNA3HA-RAP1 ΔMyb
Mybdel-F	AGCCGCAGCGGCACGCCGGGGAGCATAAGTACCTGCTGGGGGACG
Mybdel-R	CCCAGCAGGTACTTATGCTCCCCGGCGTGCCGCTGCGGCTCCGGC
	pcDNA3HA-RAP1 ΔCoil and pLAS5w-RAP1 ΔCoil
Coildel-F1	GACCGCTACCTCAAGCACCTGCGGGGCCAGGACTCAGAAACACAGCCTGATGAGGAGGAA
Coildel-R1	TTCCTCCTCATCAGGCTGTGTTTCTGAGTCCTGGCCCCGCAGGTGCTTGAGGTAGCGGTC
	pcDNA3HA-RAP1 ΔC
RAP1-BamHI-1F	GGATCCAATGGCGGAGGCGATGGATTTG
hRAP1-XhoI-289R	CTCGAGTTATTCCTCAGGTGTGGGTGG
